# "The Hospital" Nursing Mirror

**Published:** 1899-08-26

**Authors:** 


### "The Hospital" Nursing Mirror.

Being the Nursing Section of "The Hospital."

[Contributions for this Section of "The Hospital" should be addressed to the Editor, The Hospital, 28 & 29, Southampton Street, Strand, London, W.C., and should have the word "Nursing" plainly written in left-hand top corner of the envelope.]

### Notes on News from the Nursing World.

### MEMORIALS TO THE LATE SIR ALFRED ROBERTS.

The loss sustained at the end of last year by the medical and nursing professions in Australia generally, and in New South Wales particularly, is irreparable. But we learn with satisfaction of the efforts that are being made to perpetuate the memory of a large-hearted and sagacious man. His own last active work was to design and personally superintend the building of a new operating theatre at the Prince Alfred Hospital, and this is to be known to posterity as "The Alfred Roberts Theatre." A fund is being raised for the erection of a suitable monument in memory of Sir Alfred. A portion will be devoted to the purpose of presenting a yearly medal for the most successful student at the Sydney Medical School, to be called the "Alfred Roberts Medal"; and a yearly badge will also be presented to the most successful nurse in connexion with the "Prince Alfred Training School for Nurses," to be called the "Alfred Roberts Badge." By this movement it is hoped that his memory will continue to prove a yearly stimulus to those young aspirants for medical and nursing honours who, always during his life, were the special objects of his sympathy and help.

### THE DANGERS OF ORGANISATION.

In an interesting address on "The Duties and Dangers of Organisation in the Nursing Profession," Dr. George Gould, of Philadelphia, says that "to steer clear of the Scylla of a too smart independence and the Charybdis of a too decided servantship" will task the tact of the best of American nurses. Dr. Gould thinks that there may possibly be a danger lest organisation should have the tendency to tempt the nurse to try and supplant the physician, but he suspects that the "nurse's greater danger lies in the loves and hates of partisanship." Her favourite doctor, he hints, is liked altogether too much, and the one she does not fancy is not half so bad as she thinks. "It may be that she needs to rid herself of all such likes and dislikes, and fix her attention upon the impersonal aims and needs of her calling," and Dr. Gould himself has heard "of chief nurses who have turned hospitals topsy-turvy, and transformed training schools into hothouses of evil and cliqueism by assigning hated nurses to detested physicians, or by working her girls to death, and other such petty savageries." If this sort of thing prevails on the other side of the Atlantic, we hope that it is not common here.

### NURSES WITH MONEY-BOXES AT RAILWAY STATIONS.

The Portsmouth Hospital is an excellent institution, and deserving of all the support that can be afforded it. But the sight which we witnessed at one of the junctions on Saturday was scarcely edifying. A number of young ladies attired in white, with caps and aprons, and wearing Red Cross badges, were rushing about in an excited manner, pursuing passengers, and thrusting their heads into railway carriages, in their endeavour to obtain coins for their money-boxes, which were labelled "Hospital." A large proportion of them evidently did not belong to the nursing profession, some were still young enough to wear their hair down, and others were clad in elaborate muslin frocks, trimmed with many flounces and furbelows. The few nurses present were easily distinguishable by their neat appearance and their more quiet demeanour. By-standers volunteered the information that the Portsmouth Hospital was to benefit by the enterprise; but the value of the pence which some of the men in passing trains contributed was not enhanced by the fact that the money was jokingly flung at the collectors' heads.

### FEVER NURSING.

The terms of service under the Metropolitan Asylums Board are for many reasons of great interest to those of our readers who desire experience in cases of infectious diseases, and the details supplied to us by the matron of the Park Hospital, Hither Green, will therefore be appreciated. No probationers, we understand, are trained in the hospitals under the Board, but sick attendants (who are not eligible for promotion) are engaged at good wages to help. Nurses with one year's training are employed in subordinate capacities, and, after two years' experience, are qualified for the position of charge nurses. Charge nurses must have three years' hospital training, or one year's hospital training and two years' training in fever nursing. Unlike some similar institutions, opportunities of studying the different diseases are afforded to all who stay long enough. In several hospitals nurses are kept in the wards devoted to one special disorder for years. The nurses at the Park Hospital have a charming home and a delightful garden.

### THE NURSES AT THE BIRMINGHAM GENERAL HOSPITAL.

With respect to the complaints which are said to have been made by some of the nurses of this institution, the head governor observes it is remarkable that not one of them should have reached the committee or himself. Mr. Howard Collins mentions, as a proof of the popularity of the hospital, the fact that, although a premium is required from applicants, there are still more candidates than are required, albeit the number of nurses employed is 95. It has been alleged that the hard stone floors in the hospital are trying to the feet of the nurses, and this, Mr. Collins admits, may be the case; but he maintains that they are not more so than a teak wood floor, and he insists that the building is laid out to minimise labour.

### IN AID OF NURSING ASSOCIATIONS.

While the Yorkshire people have been "raising the wind" for the South Hunsley Nursing Association by means of a garden party and flower show, those of Wiltshire have been helping the Amesbury, Bulford, and Durrington Nursing Fund by holding a bazaar. In both cases sports were a much-appreciated feature of the programme, and considerably affected the attendance. The managers of the Wiltshire organisation will now be able to purchase a bicycle for their nurse. We observe, with satisfaction, the popularity this summer of all sorts of outdoor entertainments in aid of nursing associations. The increasing interest of influential country folks in their welfare is thus shown in a most pleasant and practical manner.

### A CAKE AND FRUIT FAIR.

In January, 1897, the committee of the "Wilmslow District Provident Society," realising how much of the distress amongst the poor was caused by sickness, engaged a district nurse, who soon found sufficient work to occupy her whole time. When a change had to be made last autumn the work was so well established that it was thought desirable to affiliate with the Queen Victoria Jubilee Institute for Nurses. Miss Alice Buchanan was appointed, and the whole district greatly appreciates her zealous ministrations. As the annual subscriptions do not so far cover the cost of maintaining a district nurse, a cake and fruit fair and garden party was recently held, by the kind permission of Mr. and Mrs. C. E. Ross, in their grounds of Thorngrove. Afternoon tea was provided under the trees. Stalls of fruit, cakes, and flowers were tastefully set out, and at half-past six p.m. an amusing Gymkana entertainment was given in an adjoining field. The boys' band from the Styal Cottage Homes was in attendance, and played selections on the lawn at intervals, and everyone felt that a most enjoyable afternoon had been spent. After paying expenses, a sum of over £30 was realised.

### HOMES FOR AGED TRAINED NURSES.

A movement is on foot in Manchester to start a home for aged trained nurses, and it is pointed out that there is not at present in the whole of the United Kingdom an institution of the kind. We entirely agree with the contention that nursing institutions do not answer the purpose, nor are they meant to answer it. They are intended for nurses who are able to practise their profession, and not for those who desire to retire from it. But there would be little or no necessity for homes for aged trained nurses, if during their active years nurses availed themselves of the benefits of the Royal National Pension Fund, of which our contemporary, the *Lady*, commenting on its remarkable progress, says: "It bids fair to prove one of the greatest and most successful insurance and friendly societies for women workers in the whole world."

### WORKHOUSE NURSING IN NORFOLK.

It is stated that the Local Government Board has written to the Swaffham Board of Guardians sanctioning the appointment of Nurse Campling, at a salary of £25 per annum. This appears to be a step in the right direction, and it is to be hoped the Swaffham Workhouse will benefit thereby. Unfortunately, in only too many workhouses in East Anglia the trouble with regard to the nursing of our paupers has not yet subsided, and many of the committees continue to remain powerless to cope with it. That the difficulties are not all on the side of the nurses is clear from the fact that at a recent meeting of the Norwich Board of Guardians a communication was read from the Local Government Board, enclosing a letter from an irate workhouse nurse. She complained that she was placed on night duty, and that she could not undertake the same as she was unable to sleep during the daytime. This she attributed to having spent many years in India, being the wife of a soldier. The Local Government Board had previously replied to her that they were unable to employ or discharge a nurse; subsequently, her letter was referred to the Workhouse Committee. At a fortnightly meeting of the Mutford and Lothingland Guardians, held at Oulton Workhouse, a long discussion is said to have ensued upon the question of nurses' leave. The master of the Union maintained that his was the right of granting leave, whereas the Board had decided that the head nurse should have this veto, and that leave applied for by nurses must be approved by her before being sanctioned by the Board. The master protested in a letter which was read before the Board, but the latter was obdurate.

### NURSING THE POOR IN CHESTERFIELD.

Unfortunately, Chesterfield has no medical hospital; and we agree with the mayor that considering the size and importance of the town this is not creditable to the inhabitants. Meanwhile, however, the systematic nursing of the poor is not neglected. The work done by the Victoria Home for Nurses in the past year shows that the nurse paid about five hundred free visits to the poor. On the other hand, such care has been taken to prevent the abuse of the funds that the Mayor was in a position to state that "not a penny" of the subscriptions received had been devoted to the nursing of persons able to pay. In these circumstances, the increase of subscriptions which is needed in order to secure the services of a second nurse may fairly be anticipated.

### A HOME FOR THE NURSES AT THE HIGHGATE INFIRMARY.

It is probable that the nurses of this infirmary will in a few months be provided with the accommodation which they so badly need. Subject to the approval of the Local Government Board, which it is understood will not be withheld, five houses on Highgate Hill abutting on the south side of the infirmary will shortly be purchased by the Holborn Board of Guardians. These it is intended to destroy, and on the site to erect a commodious and up-to-date home for the nurses of the institution. The modest expenditure contemplated will not excite the alarm of ratepayers, who recognise the importance of suitable provision being made for the nurses.

### SHORT ITEMS.

It is intended to close the fund having for its object the endowment of a district nurse in connection with the Cottage Hospital at Ormskirk, which is to form a memorial of the late Countess of Lathom, on September 1st.—According to the report of the Garstang and District Nursing Association for the year ending May 31st, 1899, Nurse Dell has given 20 lectures, and the nurses have attended 104 cases, paid 1,758 visits, and stayed 16 times in patients' houses. With the aid of a member of the committee a bicycle for the use of the nurse and her assistant has been purchased.—A nursing home is to be built at Pitlochry at a cost of £1,000. An evening concert given this month in aid of the fund realised £23 10s.

### Gynæcological Nursing.

By G. A. HAWKINS-AMBLER, F.R.C.S., Surgeon to the Samaritan Free Hospital for Women; Assistant Surgeon to the Stanley Hospital, Liverpool.

(*Continued from page* 269.)

THE NURSING OF MINOR OPERATIONS.

WHEN the patient has been placed in bed after a minor operation (the bed will of course have been warmed by hot bottles placed in it beforehand) she will be put on her back and covered with blankets; and if she have any tendency to chilliness or collapse hot bottles, protected by being wrapped up in flannel, will be placed under her arms and at her sides and feet to promote reaction. The nurse will sit by the patient, who lies with her head supported by a low pillow, a clean towel being placed under her chin, and a small basin or soapdish lying conveniently in case of vomiting from the anæsthetic. If there be no vomiting the patient will probably sleep, though she may have a good deal of pain; but, unless the doctor has given definite instructions, nothing will be used in the way of hypodermic injections or morphia suppositories without first communicating with him. As a rule the pain is not very severe, and the patient, so often restless, gets snatches of sleep. If there be much tendency to sickness a vinegar cloth placed near the patient's mouth will be found refreshing, and of course no food will be given until the sickness abates, after which the woman will be put on such diet as the doctor has prescribed. This usually consists of slops, tea, milk gruel, beef tea, bovril, mutton tea, milk and lime water, milk and soda water, jelly, sips of hot and cold water. If the patient be very much collapsed and faint, it often relieves her a good deal to sip a teaspoonful of hot water every minute or half-minute for eight to twelve doses.

The nurse must keep a sharp look-out for hæmorrhage, which is at times dangerous and unexpected in apparently simple cases. Unless she has been told there is no likelihood of this, it is well to observe the dressings every quarter of an hour for the first hour and then every half-hour for a few hours, to make sure there is no hæmorrhage going on. If these are more than stained pale yellow, and particularly if they be bright scarlet, with fresh blood, the nurse must at once communicate with the doctor, and in the meantime she may put a firm pad of absorbent wool over the genitals outside the other dressings, and bandage it, with the exercise of a fair amount of pressure, by means of a T bandage. She may remove the patient's pillow, and elevate her lower extremity by putting two bricks under the foot of the bed. Of course, the appearance of pallor, faintness, coldness of the extremities, sighing respirations, clammy skin, thirst, together with a quick pulse, will be at once suggestive of hæmorrhage and will call for prompt treatment. If it were a vaginal operation and no help were immediately available, the patient's condition serious, and the hæmorrhage evidently continuing, the nurse would have to disinfect her hands and pack the vagina as we have described already. But this must rarely happen. I have known, however, after a simple curetting, a surgeon visit his patient in about a couple of hours to find her bed saturated with blood, blood clots piled up on the abdomen, and the patient in a very exhausted condition, the nurse telling him as he went upstairs that there was no hæmorrhage. Such a serious accident could never happen with any reasonable care, or with a nurse who had her wits about her.

Having attended, then, to the immediate questions of sickness, hæmorrhage, and diet, there are other considerations, such as the possible use of the catheter and that of douching. After ordinary curettage douching is not commenced until a plug or drain left inside the uterus has been removed. Some doctors remove this for themselves, and others depute the nurse to do it. If it be left to the nurse she must be scrupulously careful to disinfect her hands, and to carefully clean the patient's genitals with 1 in 1,000 corrosive sublimate solution, after which she will insert her finger, feel for the strip of gauze, and pull it gently and steadily out. This is not altogether the simple matter it appears to be. Only the other day I saw a patient whose doctor had left half the gauze in the uterus for about a fortnight after the curettage, and it had become very foul, and the consequences very serious to the patient. After withdrawing the gauze I usually bring the patient over the edge of the bed and douche her gently with a solution of corrosive sublimate, 1 in 2,000, about a quart of which I permit to flow in and out of the vagina without too much pressure. It is essential that the douche and everything connected with it shall be absolutely aseptic. A fresh diaper is then put on, and the operation is over.

The douching is usually repeated once or twice daily, and if the same care is not exercised as to the cleanliness of the hands and instruments the nurse will find her patient, after so simple an operation as curettage, develop very severe symptoms of pelvic inflammation.

The practice of surgeons varies. Some keep the patient on slops for two or three days till the bowels have acted, with an aperient and enema administered on the third day, after which they are allowed to take a little fish, eggs, soup, or light puddings. Some keep a patient in bed a week or two, others permit her to rise from the fourth day onwards. All depends of course on the doctor, and on the patient. But a convalescent patient must not be permitted to get up too soon or stay up too long, and she must be assisted in and out of bed by the nurse. The douching will be continued until the doctor says it may cease, and the nature of the injection, the quantity used, the frequency of its repetition, and the duration of its continuance will be referred to the doctor in attendance.

PLASTIC OPERATIONS—OPERATIONS ON THE PERINEUM.

Some of these cases of perineorrhaphy, the repair of old tears of the perineum occurring in childbirth, require the use of the catheter; and in some it is not easy to pass it. It is particularly essential to wipe away all blood and discharge from the neighbourhood of the urethra, and it is well for the nurse to see where she is going to insert the catheter. Apart from the fact that some women urinate badly for a few days after these operations, it is well not to mess the dressings with the dribblings of urine voluntarily passed. It is usual to clean the neighbourhood of the wound very gently of blood and discharge once or twice a day, after which the wound is freshly dusted with iodoform, or iodoform and boracic acid, which keeps it dry and clean.

The treatment of the bowels is according to the methods of the individual surgeon; some keep the bowels closed for several days, until the wound is knitted fairly well; others, amongst them myself, prefer to give an enema every day from the first day, so that the bowels may act comfortably, and there will be no risk of hardened masses of fæces to put a strain on the uniting tissues. Stitches are often left in two or three weeks, or longer, until there is some consolidation of the parts. In other respects, the nursing of these cases varies very little from that of other operations in the neighbourhood of the vagina.

### OUR CONVALESCENT FUND.

IT is very pleasant to find our readers remembering the convalescent nurses during the holiday season. We have to thank Nurse Groom for her kindly gift of 3s. towards the Hospital Convalescent Fund, which is an acceptable addition to the amount in store for this good purpose.

### Holiday Experiences.

### NURSES IN THE EMERALD ISLE.

When we three friends working in a well-known London hospital learnt that our holidays were arranged for at the same time—the first week in August—many were the consultations and suggestions as to where they should be spent, that we should spend them together being a foregone conclusion.

Nurse B—— having friends in Ireland decided us to go there, make their house our headquarters, hire bicycles for a week, and in this way see a little of the country. On our arrival in Dublin, with mackintoshes, we found a glorious morning, and wished for parasols instead. Economy being an important consideration with us, we decided to see something of Dublin and its suburbs before going on to co. Wicklow, where Nurse B——'s friends lived. Dublin itself, I must confess, did not impress us very favourably, but when its suburbs were reached by the electric tram, and Kingstown, Dalkey, and Killiney burst upon our view, we exclaimed almost simultaneously, "Well! this is worth coming to see." A more beautiful place than Dalkey can hardly be imagined. It is called "Sorrento," from its great similarity to the bay of that name, and the blue sky and the blue sea, with the houses built on Killiney Hill, give a foreign appearance to the place; while the hill of Howth in the distance, with its purple shadows, enhance the beautiful view.

Arriving, in the evening, at the little station about four miles from the farmhouse where we were to stay, we found an "Irish" car awaiting us, and a very "Irish" driver. "Shure and yo're welcome, me ladies, up with you and I'll give you some fresh air," was his greeting. He had evidently been informed beforehand that his passengers would be "three tired London nurses." To Nurse R——— he remarked, "It was time you came to get a little colour nto your cheeks." When we had rested for three days at the farm (where hospitality reigned supreme, and "Oh! don't be talking about trouble, shure it's delighted I am," being the reply to a murmured remonstrance when a heavily-laden tray appeared at our bedside in the morning, carried by a girl with the typical Irish grey-blue eyes), we decided to take a four days' tour, cycling first to the "Vale of Avoca," the beauty of which one can very inadequately describe. The view from the hotel was varied by mountain, stream, and wood, and cycling on through the vale for about wo miles we came to "The Waters' Meet," where we could exclaim with Ireland's poet, Tom Moore:—

> "In all the wide world there's not a valley so sweet,
> As the Vale of Avoca where the three waters meet."

Reluctantly leaving this beautiful place we cycled on, resting often on the way, and meeting everywhere with Irish hospitality. At one little cottage by the roadside we asked or some water to drink; the woman gave us milk, and on taking out our purse to pay her, she exclaimed, "Oh! you may put that up, I'd like to meet a friend on the road myself." After some hours' ride we came to Glendalough, a most romantic valley, three miles in length, with two pretty lakes, from which the valley takes its name—"The Glen of the Lakes." The ruins of seven churches are also to be seen here, where, it is said, St. Kevin founded a monastery at the beginning of the sixth century. There is a broad ledge, too, high up in one of the rocks overlooking the lake about which a romantic story is told. It is supposed to be the place in which St. Kevin concealed himself to escape from the sight of the lady with whom he had the misfortune to fall in love! Next day, as we rode through the Wicklow Pass, our ride was for some miles through quite different scenery; instead of wooded vales, rude, barren rocks rose up on either side, giving an impressiveness to the scene until we came to the picturesque little village of Holywood, where the country changed again almost abruptly to its former wooded aspect.

Our remaining week at the farm was full of interest. We had drives in a donkey cart, and tea in the hayfields; in fact, everything was arranged with a view to giving pleasure and air to the "London nurses." All too soon our holiday came to an end, and we carried back to our work renewed health, brown faces, and the grateful remembrance of much kindness shown to us in a land where "Nature has scattered her charms with a liberal hand."

### A NURSE IN BELGIUM.

How odd foreign customs seem in illness, particularly to a nurse. Taking a rambling holiday in Belgium, I suddenly became a victim to the horrid smells which violently assail one's nose in towns like Bruges and Ghent and the older parts of Antwerp. A young Flemish lady, a casual acquaintance, had called for me to show me the sights, when suddenly, near the Cathedral, in the square where the quaint well of Quentin Mastys is to be seen, I was seized with remorseless and agonising colic. My friend led me along, in intervals of cramping pain, to the hotel where I was staying, undressed me and got me into bed, having on our way through the courtyard despatched a messenger for her own family doctor. Very soon the doctor appeared, asked a torrent of questions in Flemish unintelligible to me till my friend came to the rescue. Then he promptly apologised in French, and in French we conversed until I left Antwerp, when I found that he could speak English, though to a limited extent. In silence he took out a pocket book, wrote his prescription, sounded the electric bell, said something in Flemish to the chambermaid (who came in answer), which immediately brought a "commissionaire." The doctor gave the folded paper to the man, directions in Flemish to the chambermaid, and then left us for a few hours. The chambermaid reappeared, a roll of white linen hanging over her arm, a long-handled copper frying-pan in her hand containing a steaming mixture of linseed. Seating herself in a chair by the bed, she proceeded to spread a large abdominal "cataplasme," clapped it briskly upon me, covered me up and left. Suddenly, after a tap at my door, the blue linen coated commissionaire re-entered with a bottle of physic, took off its wrapper, shook it up, uncorked it, poured out a dose into an enormously heavy tablespoon, and even put the spoon to my mouth, smiled, bowed, and departed. Poultices and physics were administered all day, the brisk little doctor came and went, my friend had a bad sick headache and refused to go downstairs to a meal, but ordered a tray of food brought up to her. Lobster salad and coffee! Scarcely a suitable meal for a bilious headache. The gentleness of the Flemish demoiselle I shall not soon forget, nor the kindly faces and manners of my doctor, chambermaid, and blue-coated hatless porter. When in about a fortnight I left my bed, the doctor recommended me to go and sit out of doors all day long in the Zoological Gardens, where he said I could buy the milk, which was still my only food, and I was to be sure to take my big bottle of medicine and the massive spoon with me. So far I obeyed strictly, but when it came to drinking milk I rebelled, for I found that it was to be had only in the combined establishment for pet cows and monkeys, where stood a row of beautiful patient cows each in her little stall with her name above it, and wire cages with monkeys all round amongst the plants. The combination of odour, delicate cows' breath overpowered by monkey, gave no appetite for milk.

### In a Hospital Garden.

BY A MATRON.

THIS particular garden is somewhat of a wilderness, which characteristic, indeed, constitutes its chief claim to beauty. From the ward windows at the back of the hospital you see first an unkempt box hedge forming a background for some ancient beehives, a clump of outhouses smothered in ivy—not the ivy of clinging graceful tendrils, but of strong boughs and clustering pale-green branches—and a few sapless yew trees where thrushes and green linnets nest year after year. Beyond the box hedge, on one side of the path, is a large plot of ground given over to the cultivation of homely vegetables; but on the other side lies a stretch of grass dotted with fruit trees, gnarled and twisted by age and wind and weather. The lawn is bounded on the west by waving limes and lilacs, and on the south by great trees, where in June nightingales make merry music not only all night but often all day long; and away yonder are fields and hedges, while in the distance is a silver streak that Molly thinks the road the angels walk, but other people call the river. The garden, although secluded, is never silent; with the earliest hint of daylight begins the din of all the small birds and the chatter of the starlings. At dusk you hear the subdued twitter of some late home-comer as he settles down on his pet branch, the hum and buzz of the cockchafers, or, as they are called here, the "midsummer daws," and the weird sound of bats as they whirl and circle in the gathering darkness. Through the open windows steal the low throat notes of the nightingale; at first he seems afraid to let himself go, but suddenly he bursts into an exulting, triumphant love song, which must surely send a thrill to the heart of his little brown mate. No wonder the other birds are silent when he is singing—only the night-jar, who is self-satisfied and impervious to criticism, tries to drown Sir Nightingale with his monotonous voice.

This hot afternoon Nurse Alice sits on the grass with her sewing, and Molly, who in the hospital has been very near death, alternately plays with the kittens and teases a merry-faced boy who lies on a mattress under the biggest apple tree. Being a temporary cripple, he can only retaliate by seizing his crutch and assuming a ferocious expression, which has the effect of sending Miss Molly into fresh peals of laughter. In the apple tree is Timothy, the brown owl, swaying in the wind as it lazily rocks him to sleep and ruffles his downy feathers. Yesterday, just in the middle of a delightful doze, Timothy was "mobbed" by half a dozen aggressive swallows, and finally knocked off his pet branch—a dreadful shock to his dignity. The bees swarmed this morning, and in hiving them from the yew tree they chose to settle on, the green linnet's nest was rudely shaken, and her babies fell out into the very jaws of the kitchen cat, and so her always plaintive note is still more sorrowful—Rachel weeping for her children and will not be comforted. Molly, who at six years old is a little person of infinite resource, proposes that Nurse shall explain to the disconsolate mother that the little birds are gone to heaven, and is highly indignant when her suggestion is greeted by a titter from Boy Alfred, as she patronisingly calls him, though he is her senior by twelve years or more. A scene seems imminent, but Nurse tactfully suggests that Molly tells them one of her pretty stories, which she is nothing loth to do, and forthwith relates how a dear little baby was put into a basket among the bulrushes, and how, when the basket was opened, the baby "began to sing." Peace being restored, Nurse goes to the other side of the lawn, where is another bed, and stretched out on it a thin white-faced lad, who looks as if he has scarcely energy enough to turn on his pillows. He is a Londoner, now in the country for the first time. He came down to execute a commission at a neighbouring great house, was taken dangerously ill, and brought into the hospital. Rural sights and sounds are a novelty, yet he takes but a languid interest in all that is going on around him. He is considered by his fellow-patients to be rather "high-minded," but the fact is his brain contains but a single idea—that of being one day a great artist, and he bitterly resents illness and suffering, or any obstacle that comes between him and his goal. His face brightens as he sees nurse coming across the grass, for when he talks of his art pain is forgotten. He tells of hopes and ambitions, of the power that he feels is his, of how he intends to fight poverty and want of influence, and some day to "come to the top," and nurse smiles cheerfully, and answers hopefully, though she knows that a few short months will bring farewell to fair dreams and all youth's goodly heritage. It seems unnatural to think of death this perfect summer day, with life everywhere around, but his shadow falls very near us, and we who know it is there keep smiling faces and joyous voices till such time as the shadow falls more darkly, and is beheld by the one who has been longest ignorant of its presence. Then by Nature's kind provision it often seems not the shadow of a dread evil, but of some glorious angel. This Nurse Alice knows, and the knowledge helps her to repress the sadness that must else rise to the surface, and rudely betray to the boy the secret God has not yet seen fit to whisper to him. In truth, "All our life is mixed with death, And who knoweth which is best?"

There is the sound of a bell, a shriek of "Tea!" from Molly, a sudden scamper of kittens down the garden path en route for the kitchen, and another afternoon is over.

### A Doctor's Word Portrait of a Nurse.

IN an address recently delivered on "Home Nursing and Hygiene" to the members of the Glasgow Sick-Poor Nursing Association, Dr. J. A. Wilson, alluding to the nursing of the poor in their own homes, observed: "We usually say that a stranger makes a better nurse than a relation, for the latter lacks authority, her hand may be restrained by her heart, and her judgment perverted. A nurse is a trained, intelligent woman, whose occupation is trying, though benevolent and honourable, and she has to deal with people who are sick and suffering, and may be anxious, petulant, or despondent. She must have self-control, cheerfulness, patience, and she must be tidy and clean. There is a variety of nurse who becomes inflated with the importance of her services, and who wears so much 'side,' to use a vulgar term, that she makes herself an insufferable nuisance in a house."

### Swaffham Cottage Hospital.

THE supporters of this hospital held their eleventh annual meeting last week. The hon. secretary, Mr. A. J. Winter, kindly allowed the meeting to take place in the grounds of Sydney House. Mr. Winter, who read the report, said that the hospital was again in a satisfactory financial position, there being a balance in hand of £24 16s. 4d. after paying all expenses for the past year. The hon. secretary went on to say:—"The committee have to report to the subscribers that in May last Nurse Geldart tendered her resignation as matron, and that the committee accepted the same, and have engaged Nurse Harwin, who has been a nurse in the Lynn Hospital over two years, to succeed her. The thanks of the committee are tendered to Nurse Geldart for her services rendered during the past seven years."

### Across the Seas.

### NURSING ON THE WEST COAST OF AFRICA.

About eighteen months ago I was offered the post of sister in the Colonial Hospital at Lagos, West Africa; and as I dreaded the approaching winter and longed to get away to a warmer climate I accepted the offer eagerly. My friends were rather alarmed at the idea of my working a whole year in that unhealthy climate, but as I have spent the greater part of my life in the tropics, I considered I was on sufficiently intimate terms with the much-dreaded malarial microbe to defy him. After the usual preliminaries had been gone through with the Colonial Government, I sailed from Liverpool about the end of October in the ss. "Angola," which at the start was fearfully overcrowded. In three days we were basking in brilliant sunshine, and as a large contingent of passengers left at Madeira and some more at Canary, everyone was made comfortable for the rest of the voyage. Reading, a little needlework, deck quoits, and concerts on deck in the evenings after dinner helped to while away the time.

We steamed into Lagos Roads at sunset, the passage lasting exactly a month. On my arrival I felt well and strong, and capable of battling with all the malarial microbes in creation. I did not remain in this blissful state many weeks though; the wily little wretches soon claimed me as a victim, and I did not get much sympathy from my fellow-workers, as I had been holding forth somewhat boastfully on my "tropical experiences," and they only laughed unfeelingly at me when I went down with my first attack of West Coast fever. Having anchored in the Roads, I naturally concluded that my journey was over, but the worst part of it was yet to come. A formidable row of breakers lay between us and Lagos town. The liners are too big to cross the bar, so branch boats of much smaller build take the passengers over, and the heavy luggage goes up by the Forcados River, where it is transhipped to the branch boats and brought down to Lagos about a week later. There was a terrible surf on, and a little cockleshell of a boat, which was to convey us to the branch boat, was bobbing at the ship's side, one moment on a level with the ship's deck and the next down in the depths. The branch boats are not allowed to cross the bar after six p.m., and as they are not provided with either food or sleeping accommodation for passengers, Captain Goudie very kindly ordered dinner an hour earlier for us on the "Angola." By the time dinner was over darkness had descended on the face of the waters, and the transhipping had to be done by electric light on the one ship and very nearly total darkness on the other. The male passengers scrambled over the ship's side and hung on to the rope ladder, waiting their opportunity to let go and drop into the boat. I wondered if I was expected to do the same, as I saw no other means of accomplishing the descent, unless they hoisted me in bodily by the crane, which was getting the passengers' luggage into the boat. Presently a shout was raised for the "mammy's chair." The "chair" was a cask sawn in half with a rough plank across it to serve as a seat. Ropes were fastened to it on three sides and hooked on to the crane. The seat was draped with a Union Jack, and the "white mammy" was invited to mount her chair of state. "Let go!" shouted the captain, and I was swung out into space for a moment, and came down with a terrific bump to the bottom of the boat, and hopped out "one time" as the Kroo boys say. The captain of the branch boat was good enough to place his comfortable cabin at my disposal, and next morning invited me up on the bridge, where I could get a fine view as we crossed the bar. Lagos looked very pretty and picturesque in the early morning sunshine, the clear blue lagoon reflecting the clouds and palms. The sanatorium was pointed out to me—a little white house with the lighthouse peeping out between the trees, and just a glimpse of the sea and a strip of white sand lying beyond. The place is kept up by the Government, and officials are sent there to recruit after illness. I did not think much of it myself as a sanatorium. It is very hot, and abounds with sand flies and mosquitoes. It has since been condemned, I think, by the chief medical officer. I was met at the landing stage by the matron and chief medical officer, and after struggling through a clamorous, perspiring crowd of blacks we managed to reach the "rickshaws" which were waiting to convey us to the hospital. The latter is built in detached blocks, scattered in a large compound which has since been enclosed by a wall. Each block consists of one large ward, two small rooms, used either as private wards or bedrooms for the native male nurses, a lavatory, and a scullery. They are built of wood, and are raised about twelve feet from the ground on wooden pillars. They are each surrounded by a wide, shady verandah, and the space underneath the wards forms a cool promenade or lounge for convalescents. The blocks are numbered alphabetically. A and B are set apart for European patients, six beds in each. The native male wards C and D are bigger, having sixteen beds each. The accommodation for female patients was very bad indeed, and the female nursing staff most inefficient; but when I left Lagos six months ago a new block was being built, which was bigger and better situated than any of the others, so the poor black mammies will be the best off now.

The Europeans chiefly come in with fever or dysentery. A combination of the two is to be dreaded, and generally means invaliding home, or to Canary, if the patient is lucky enough to recover. We had only one surgical case in the European wards during the year I was there—a compound fracture of the femur—which did very well, though at one time we were afraid tetanus was setting in. I may as well say that we had only two deaths of Europeans in hospital in the twelve months, but I am told it was an exceptionally healthy year. One of the patients who died came in with malarial fever. He had been treated outside by a private doctor, and was brought into hospital as a last resource. His temperature was 105 deg. on admission, and varied between 102 deg. and 105 deg. for a week. It then went up suddenly to 107 deg., and for nearly twelve hours it oscillated between 107 deg. and 109 deg. We gave him three ice baths, each time lowering his temperature to about 104 deg. Unfortunately, the supply of ice was soon exhausted, and so was the patient. He died about three a.m., with a temperature of 108 deg.

The native male wards are chiefly filled with ulcerated legs and toes, caused by guinea worms and jiggers. Nasty little things the jiggers are, too. They are scarcely bigger than a pin's point, you can hardly see them, but there is no mistake about feeling them. The natives are rather clever at extracting them. Cases of tetanus are very common, and in the rainy season pneumonia prevails to a great extent among the Kroo boys. They are very unsatisfactory cases to nurse, and nearly all the patients die, just because they make up their minds to die. Medicine and food are both taken under protest. "Why you trouble me, I no live, I live for die," and die he does sure enough.

### To Nurses.

In order to increase and vary the interest in the *Mirror*, we invite contributions from any of our readers in the form of either an article, a paragraph, or information, and will pay a minimum of 5s. for each contribution. All rejected manuscripts are returned in due course, and all payments for manuscripts used are made at the beginning of each quarter, *i.e.*, January 1st, April 1st, July 1st, and October 1st.

### Hospital Secrets.

As Told by a Steward.

### DAN'S DISAPPOINTMENT.

Dan came to the Royal Central West Hospital as a patient. Something ailed the toes on one of his feet, and a quack doctor who lived near at hand in Islington, and to whom he had gone for advice, had been clever enough to see that herbs were of no use and that a surgical operation was necessary. He therefore recommended Dan to seek admission as an in-patient at the hospital. "Wot'll they do to me? I won't let 'em cut me foot orf, if I knows it," said Dan. The vastly superior knowledge of the quack provoked him to reply, "No; you mustn't let 'em do that. Keep yer wits about yer, and directly they knows that ye're watching the job they'll pass on to the next bed. I never sends my patients to the 'orspital when I ken 'elp it, but yer see yourn is a case as needs pertickler treatment. These 'erbs as I've got ere would make yer blood all right, but they won't make the joints in yer toes straight, see?"

So Dan came to the hospital. A couple of porters carried him from the out-patient department in an invalid chair to the lift, and from the lift to number seven bed in Munster ward. Dan was quickly walled in by a screen, and quickly undressed, and quickly washed, and quickly put between some ready-warmed and comfortable sheets. When the screen was removed, and Dan had somewhat collected himself, he looked about and found that he was one of a score or so of male patients in one long ward. Presently Sister Munster approached him, and, affectionately addressing him, said: "Well, dad, have you ever been in hospital before?" Dan did not answer for a moment. He was too astonished by the easy assurance of the kind-eyed Sister who styled him "dad" before he had had time to say, "Halloo, there!" When he at last found his voice, he replied with trepidation: "No, I ain't, miss, thank yer." "Ah! well, you'll very soon be well again," said the Sister. And, after smoothing the pillow and tucking in the bed-clothing at the side, she turned to speak to number six, who was another new arrival.

Dan was with us for nearly six weeks. A few days after admission he was taken to the theatre, and his toes were most successfully operated upon. Ten days after the operation he was allowed to get up for a little time, and sit with the circle round one of the big fire grates. His conversation proved to be more interesting to his fellow-patients than did that of any of the others. It appeared that until recent years he had been all his life a fairly prosperous Punch and Judy man. He had never been a man of means, as he put it, because he "had not had enuf eddication to accept privit engigements, as some in his honored perfession did for two or three quid a time, and he had never been lucky in getting a honest pal as would pliy good musick and deal strite with the ouf in front whilst he was hid up in the show." But in his palmy days he had knocked along very steadily with his three or four pitches a day in North London. When the weather had been favourable, and trade had been good, he often went home at night with eight bob in his pocket. On Saturdays he had sometimes knocked the figure up to twelve bob. If he hadn't married the gal he did—though he didn't wish to say anything against her, for she had died two years ago—he would have had more than enough to live on now. His wife would have been a good sort if she would only have kept orf of the booze. Before coming to the 'orspital he had been able to square his rent up and to give the landlord's man enough over to ensure the room being let in his name for several weeks to come, and for a consideration he had put his faithful little dawg Billy into the safe keeping of a policeman's wife who had no children, and whom he had known since she was a nipper, and whose husband being on night dooty, found the darkness not quite so lonesome like when the little dawg was a-sleeping on the mat at her bedroom door. Dan's lameness, which had been coming on for years, had greatly interfered with his duties. Latterly he had only been doing two pitches a day, some days not even that, for he could not stand much, and the fixing up and taking down of his show seemed to exhaust him. In his early married life he recalled how he had done a splendid day's work on the Saturday, enjoyed a restful Sunday, been up betimes on the Monday, put his show on to the top of a cab and driven to the railway terminus, booked to Margate, worked hard there until the Friday evening, and then been back in town again for the rich harvest of Saturday. But all such rushing about, with the increased toil involved, had long been out of the question for him. Various circumstances had made it impossible. Once on his return on the Friday night, for instance, he had discovered that his missis had pawned nearly all the things in the little home for booze. There was none of the wife-beater about Dan, and he had borne much for many years in silence. He used to think that when physical infirmity did overtake him the weak part would be his throat, and yet, as he pointed out, it was at the part of his person farthest removed from his throat—his toes—that the ailment lodged.

Sister Munster came to name him Docile Dan; he was so patient, so well-behaved, so ready to be of service, so eager to avoid giving any trouble, and so cheery and hopeful. He was persuaded that his foot was getting as strong as ever it was, and he became more and more anxious to give proof of his gratitude.

On the eve of the day of his discharge he walked up to the Sister's table, and touching his grey bush of front hair, he begged to be permitted to make a suggestion to her. The idea, he explained, had occurred to him as he lay in bed the previous night. He hoped Sister would believe in it, and give or procure the necessary permission for the carrying out of the project, which was neither more nor less than that he might be allowed to bring his show, with his champion little dawg Billy, to the 'orspital, and give an exhibition of their combined powers in the children's ward. And he wanted Sister Munster to be so good as to square the secretary, or the matron, or the chairman of the managing body, or whosoever it might be whose sanction was indispensable to the proceedings. And Sister Munster did approve. She saw the matron, and that kind lady interviewed the secretary, who expressed admiration at the old patient's generous impulse, and unhesitatingly granted the request.

Dan cried tears of pure joy on calling here two days after his discharge and learning from Sister Munster that the authorities had been pleased to accept his kindly-offered entertainment in the children's ward. He swore that it would be the proudest day of his life. He went his way, and had only half a supper in order that he might give twopence a yard more than was his custom for the scarlet material with which he draped the lower part of the wooden framework of his show. His excitement was almost child-like; it prevented him from sleeping soundly, and he dreamt that he could already see the happy children and hear their merry laughter and shouts of glee as he made Judy hit Punch a dozen times over the head with a heavy stick.

When morning dawned he washed little Billy with warm water and a penny piece of scented soap, purchased specially for the purpose. He put some new brass pins round the front shelf of his show, and otherwise touched his property up with a view to giving heightened satisfaction to his patrons at the 'orspital. Then, before dinner, he and Billy went most conscientiously through a full dress rehearsal.

As two o'clock struck, Dan presented himself at the hospital gates with his show on his shoulder and Billy at heel, wearing a muzzle. Once in the children's ward, and Dan had more little hands to assist him in erecting his show than he could control.

But the performance began at last. And what a performance it was! Each item seemed to be encored half a dozen times. The little sufferers who could not leave their beds cried out for clever little Billy to be carried to them to pat. Poor Dan was hid from sight for three whole hours at least, and it was only on Sister Munster's urgent appeal that he brought his display of talent to a begrudged close. The ward was crowded. Juveniles and convalescent cases from all parts of the building had been brought to share in the treat. Students and sisters and nurses, and actually two members of the staff, had wandered in, and were to all appearances as delighted as those for whom the performance was provided.

When Dan finally emerged from his show with heated brow and a happy gleam in his eye Sister Munster handed him his cap half filled with copper and small silver. The moment he saw it the triumphant smile left his face. He pushed the cap with its contents away from him. The well-meaning Sister failed to understand the situation and insisted on his acceptance of it. Taking it, he emptied the contents into his red handkerchief, and tying the corners together he put it and the cap solemnly into his coat pocket. He took his show to pieces, strapped it together into a carryable load, lifted it silently to his shoulder, and made for the door of the ward without a word of farewell. Sister Munster endeavoured to stop him and pressed him to stay and take a cup of tea, but no, he would not. He gained the quadrangle and reached the front gates. There he put down his burden, took from his pocket the red handkerchief, untied it slowly, and then, a few at a time, he dropped all the coins into the contribution box that is a fixture on the side wall. Again lifting his burden to his shoulder, and without one backward glance, he trudged sadly away followed by Billy.

Dan has not forgiven us for wounding his generous feelings, and we have not forgiven ourselves.

### Minor Appointments.

Victoria Cottage Hospital, Emsworth.—On August 11th Miss Florence G. Hughes was appointed Nurse Matron. She was trained at Guy's Hospital and Lambeth Infirmary. Subsequently she has been probationer and staff nurse at Lambeth Infirmary, probationer and head nurse at Guy's Hospital, and Queen's probationer and Queen's district nurse.

Southport Infectious Diseases Hospital.—Miss E. Downing has been appointed Charge Nurse at this hospital. She was trained at the Batley and District Hospital, Yorks, and afterwards for four years held the post of charge nurse at the Huddersfield Infectious Diseases Hospital.

Metropolitan Convalescent Institution.—Miss E. A. Brander has been appointed Assistant Matron at this institution at Walton-on-Thames. Miss Brander was trained at the Royal South Hants Infirmary, and acted as staff nurse at the Birmingham and Midland Hospital.

Royal Hospital, Belfast.—Miss Annie Dane, who was trained at the Western Infirmary, and is at present charge nurse at the Fountain Hospital, has been appointed Night Superintendent at the above institution.

Carnarvon and Anglesey Infirmary, Bangor.—On August 7th Miss Laura Charles was appointed Charge Nurse of the women and children's wards. She was trained at the same infirmary.

### Night Nursing Extraordinary.

By a Nurse from a County Lunatic Asylum.

I often wonder if an energetic woman could be set a more impossible task than that of being absolutely idle for the greater part of eleven hours. Yet this is what is expected of the night nurses in most, if not all, of our county lunatic asylums. No one acquainted with the work will deny that it needs a woman of more than usual energy to fill a post as mental nurse, or, as she is more often called, attendant. By the way, why should one who ministers to the mind disease be less a nurse than one who ministers to the body? This nurse, then, goes on duty probably at eight p.m., and leaves it at seven a.m. as a rule. During that time she has to "peg the clocks" every half-hour—most asylums being now obliged to have the electric or other clocks in every dormitory—and if she has cases of general paralysis there will be these to attend to and change about every two hours. The dormitory is in darkness, unless the tiny ray from the lowered gas jets can be called light. It is more a means of making darkness visible. She has a bull's-eye lantern in one hand, and having visited single rooms, examined suicidals, and attended to the general paralytics, she sits down, to take up her work or a book one would suppose. But no, this is strictly prohibited. Her duty is to sit and stare into darkness, so that, forsooth, she may not become too absorbed in what she is doing to be ready in case of necessity. Another half-hour goes. Lamp in hand she walks round her dormitory, pegs her clock, and returns to her seat. It may be that absolute quiet reigns, in which case it is doubly hard not to be overpowered by the inclination to sleep—dread temptation—but that depends greatly upon the class of patients. If they are of the acute maniacal type there will be shouting and singing perpetually. They may be suicidals, in which case constant alertness is necessary to detect the slightest sound, and as this class is generally the most quiet it is the more difficult. Then again, in the infirmary wards, what with medicines, treatment, feeding, changing, &c., the time flies as in ordinary sick nursing.

The arrangements for the sleeping accommodation of night nurses still leave much to be desired, even in these enlightened days. One night nurses block will be built next to the laundry. Another asylum will have no separate block at all, and the night nurses then have the pleasure of listening to their next door neighbours' energetic proceedings on either side when they happen to be off duty or up to tidy or to fetch some forgotten thing or other during the dinner hour, and so on, *ad lib.* There will be corridors being polished, rooms turned out (one or two every day), and our poor night nurse turns herself on her other side and wearily thinks, "No peace for the wicked," or else, "A night nurse's life is not a happy one," then gradually sinks off to slumber again, only to be awakened afresh by some new terror in the way of noise. After eight hours or so of this she is expected to be fresh for duty and for eleven hours of darkness.

If only the wise men of an asylums committee would but try it just once they would learn not to ask impossibilities. It would be better still to follow the example set by poor law guardians and appoint some practical common-sense ladies, who would soon point out the error of such ways. To a woman brought up to use her hands at all it is impracticable, and tends to lower still more the tone of a system of nursing already notorious for the class of people who alone can live among such surroundings with rules which only breed deceit.

### "BARBADOS."

If the writer of a communication on this subject will send her name and address to the Editor (not for publication, but to authenticate her statements), he may be able to make use of it.

### Echoes from the Outside World.

### AN OPEN LETTER TO A HOSPITAL NURSE.

If ever "Sunny Southsea" merited its name it does so this year. The whole place is bathed in sunlight, the sea sparkles and shimmers, the glass roofs here and there catch the reflection and throw back bright rays again, and yet there is always a cool, refreshing breeze which prevents our being overpowered by the heat. Just in the middle of the afternoon we stop at home and read, or work, in preference to facing the glare on the parade, but that our idea of pleasure is not universal is proved at once by a glance towards the beach any day after lunch. There are the children still paddling; their mothers, with their backs up against friendly boats, working, reading, or mending; the white-robed nurses having a chat with a friend or an admirer, while their charges collect buckets of stones; the men reading the papers and smoking, or, stretched at full length, with hat tilted over their eyes, trying for an afternoon doze on their stony bed. Vendors of toys, fruit, or cakes appear now and then, a couple of performing dogs, or a monkey troupe gather around them a large crowd, and reap a correspondingly large harvest of coins. Everyone I meet looks in the rudest health, and no wonder, for the visitors to Southsea live out of doors more than most people, because so many entertainments are provided for them in the evenings, as well as during the day, that they are enticed out again directly the dinner or high tea is over. Just now "The Musketeers" are attracting large crowds. The troupe consists of four men, said to be part of the chorus of "The Greek Slave," three of them dressed in the voluminous cloaks and sombrero hats with which the stage representations of Dumas' novels have made us familiar. They also wear black masks, except the comic man, who, wisely remembering that play of feature materially helps to excite risibility, does nothing to hide an important part of his stock in trade. Besides the comic element, which is capital, the four men sing glees and quartettes, and contribute solos which more or less delight their audience.

---

If you want a holiday in a lively place, with good sanitation and a splendid air, easily accessible from Waterloo, you will find that Southsea suits all pockets, tans all complexions, and drives away that horrible feeling of "tiredness" in a few hours. As to excursions, no one need be dull for a day for the want of something to do. In addition to the harbour and the dockyard at Portsmouth, there is the Isle of Wight so near that an expedition over the water costs little in either time or money, and this clear weather the coast, with its fringe of trees down to the water's edge, seems most tempting. The boats go several times a day to Ryde and Cowes, Bembridge and Seaview. The latter is the more trying passage to those who do not like any movement on the water. The day we went several ladies and many children began to wish they had remained on terra-firma. But I thought it lovely, and my second visit to Seaview quite confirmed my original ideas on the subject that it is a dear little place, and most picturesque, with its many-coloured tents erected on the sand along the seashore and the pretty wooded slopes behind. There appeared to be an epidemic of bare legs the day we were there. The children were all uncovered, and even some of the ladies were going about in shoes, minus stockings; but in many cases the fact was hardly noticeable, for the legs were so tanned that they appeared as if covered with brown hose. Men, women, and children all wear the same hats this year everywhere I find —a white linen hat, rather like a man's bowler, with a wide brim. The advantages of these hats are many. They are beautifully light, cool, and shady, very becoming, and very cheap. Children cram them on at all sorts of funny angles, and look delightful in them.

Any novelty in the way of a holiday is always eagerly sought after, and when, at the beginning of the summer, my attention was drawn to an announcement that one of the Orient Company's steamers intended to proceed to Spitzbergen with pleasure passengers, I had an intense longing to join the party. These holiday makers, 230 in number, have just come back from their trip, and, furthermore, have returned in safety. Consequently, next year there will probably be three or four expeditions in the place of the one which started this summer. But it appears that such a voyage is attended with considerable danger, because the waters around these Polar seas are practically uncharted, and the perils which a large iron vessel of several thousand tons must run from sunken rocks and floating ice are very great. The meeting with "Polar pack ice,"—which is spoken of as one of the experiences much to be desired —might possibly mean disaster to all concerned, for in case of a catastrophe the vessel would obviously have to depend entirely upon its own resources for repairs, docks and workmen being far removed from these regions of snow and ice. Therefore, much as I should like to be able to spend a holiday in such a delightfully original manner, I think I shall wait a few years till navigators have learned rather more about the ins and outs of 80 deg. north latitude, though I must beware, I know, of deferring too long, lest I should only arrive at Spitzbergen with the landscape advertisements of patent Pills and comforting Cocoa.

---

Have you read a book called "The Domestic Blunders of Women"? The writer, though he dares to make the assertions, does not dare to admit his identity, lest we turn upon him and rend him, so he contents himself with the signature of "A Mere Man." Some of the statements would incline a mere woman to fancy that he has never had any genuine experience of matrimony, but is a clever bachelor of an elderly type, who has had to make a fetish of his cynicism for the want of someone else to love and admire. He declares that a woman knows nothing of the value of money. Possibly, of course, he may be right, but if the average man were given half-a-crown to buy a lunch for his family, and his wife were allowed the same sum, I think I can guess who would provide the best and most appetising meal for the money. Also, I have frequently noticed that if the pence are to be saved, and economy is to be the order of the day, it is not the man who will undertake the task. The average husband will say, with much nobility, "All right, my dear, if it must be, I suppose it must, but for goodness sake don't worry me as to the details. I don't mind what you do as long as you leave me my pipe and peace." And then when round of beef comes to table in the place of sirloin, the same magnanimous individual "can't eat such rubbish as that." "The Mere Man" in the book asks a strange question, "Why is domestic service the only profession or trade in the world which is overstocked and detested?" Detested it may be, but that it is not overstocked "The Mere Man" would soon find out if he needed a general servant, or even a good plain cook. He would be met by the reply from the registry officer that "lots of ladies have been waiting a month or two for a servant," and that they daily become scarcer. One more point I must touch on. "Most wives owe any health they may have to the persistent interference of their husbands." I had an idea that in most cases it was the wife who endeavoured to dissuade the dyspeptic husband from eating indigestible food, or the man with a weak throat from over-smoking, but these cases have evidently escaped the writer's notice. I wish he had chosen a subject about which he had known rather more, or treated it a little more fairly, then I should not have been obliged, in defence of my sex, to take up the cudgels and seem to be abusing the men. In simple justice I am bound to admit that in many ways they are still distinctly superior to women, but in housekeeping as yet they are the merest tyros, and there is no good their pretending that they are not.

### A Book and its Story.

### MR. W. H. MALLOCK'S NEW NOVEL.

MR. MALLOCK'S recent novel* is a brilliant satire on the schemes of *fin de siècle* social reformers and would-be philanthropists; the foibles of lovely, frivolous, and yet withal shrewd, society women; and the new woman on her war-path, holding crusade against all existing means of alleviation of the woes of her unfortunate sex. It is all so clever that it is to be regretted so many good things are compressed between two covers. For taken in moderation how stimulating good things are; how exactly the reverse when the feast is more than can be comfortably digested at once. By this it will be understood that "The Individualist" is a book not to be hurried over, but to be kept by one and read with leisurely dignity, if anyone has time to treat an author thus in these hurrying days.

Mr. Mallock excels keenly in analysis of character, and in his writing there is also that rare faculty of suggesting, without exhausting thought; one listens not only to what is said, but under all there lies, to those who can hear, the sound of what is still unsaid. The character of Tristram Lacy, the hero, is a fascinating one. It was at Oxford that he first came into real contact with the criticism and scientific thought of the day. His brief experience there influenced the whole of his future career. Until then, religion had been to him a refuge amid the inward storm and stress to which he spiritually was naturally no stranger, and to it he had betaken himself as to a heaven in times of conflict and distress. But "he found in the lecture-rooms that tutors treated St. Paul not only as if they did him too much honour by mentioning him in the same breath with Plato, but even in the pulpit of his lately-restored college chapel surpliced critics would undermine in reverential whispers the very foundations of the faith which it was ostensibly their duty to defend. Contact with these new views was at once a shock and a stimulus to him." It aroused all the latent energy of his speculative, critical mind, always on the alert to inquire, and ever difficult to satisfy, even when proof was not lacking to confirm the evidence presented. He studied "Roman Theology," hoping there to find a rock at whose feet the waves of doubt and scepticism would be laid to rest as in the arms of a patient and indulgent parent; but even here, whilst fascinated by its arguments, he failed to be permanently influenced by its teaching, and so he drifted on the sea of religious doubt, finding no anchorage in any of the forms prescribed. "Profoundly impressed by the sublime mysteries of the Christian worship, he became gradually to disbelieve in everything that disassociates the human destiny from the destiny of the dust." The unhappy effects of agnosticism are seen in every action of his life. A certain mental *malaise* weakens and disturbs the inner life, while outwardly this is apparent in a constant change from one pursuit to another. With a brilliant intellect, and all those physical gifts which go to make a man, with some of the material disadvantages in his earlier manhood which help to make a hero, he yet, when later the possessor of wealth, which had hitherto been lacking, failed to find life in any degree worth the living. His astute, and worldly old uncle, Lord Runcorn, made a perfectly correct diagnosis of the cause of the *ennui* to which Tristram Lacy was a victim when he described it as being produced by neither Love nor Liver, but as having its roots in the intellect altogether; in other words, he was the victim of "a disease produced by a sane and unimpaired logic working on such premises as are supplied to it by contemporary philosophy." He was merely suffering from "the effects of the doctrine of negation pushed to its full logical consequences. . . . Every promise of distraction, every pursuit and ambition stimulated him only till its first rewards had been tasted by him." Then he at once realised the vanity and vacuity of the things which had so lately charmed and delighted him.

Much entertainment will be derived from the description of Startfield Hall and its promoters. Lacy, when on a voyage of discovery to find its whereabouts, is one foggy evening lost in Bloomsbury, not far from the vicinity of the hall itself, and ultimately finds himself, in response to an inquiry of a stranger whom he meets, in the presence of Mr. Prouse Bousfield, its genial, if erratic, supporter. He dines with him and is introduced to "one of the most famous women in Europe, Mrs. Norham, *the* Mrs. Norham, the authoress"—and social revolutionist. It is a very amusing scene, particularly so to our hero. The hospitable host and his little purry, adoring wife, with her knitting and inconsequent platitudes breaking in at intervals upon the "advanced" and serious theories which are agitating the brains of her guests in a manner which might have been pathetic, but is, in consequence of her unconsciousness, ludicrous. The earnest, stentorian tones of Mrs. Norham, airing her views; the awe-inspired host, and the courteous, high-bred Lacy receiving quietly the astonishing doctrines propounded by Mrs. Norham, who, however little difficulty she has had in solving existing social problems to her own satisfaction, finds herself suddenly uneasy under the polite and critical gaze of the stranger. Apparently he belongs to a part of the community to which she is personally a stranger, although it is part of her principles to aim at the destruction of those who form it. Lacy asks to be informed of the object which she has at heart. If it meets with his approval he might assist pecuniarily, as the proposed hall is to be erected on a site of which he is ground landlord. "Mrs. Norman again stared at him. The rich were amongst the objects of her systematic satire, but at the thought that her neighbour might be one of them her manner instantly softened."

"'Our aim—my aim—for the movement is really mine, though other brains more powerful than mine are helping me—is to elevate . . . . that great, blind, dumb, helpless, unthinking, piteously-brutalised multitude, by whom,' continued Mrs. Norham, who, satisfied that the stranger was watching her, fixed a rapt look on the cornice, 'by whom,' she continued slowly, 'all the greatness, all the wealth, all the comforts, all the refinements, all the luxury of this and all other countries are made.' 'You should surely add,' said the stranger, with courteous gravity, 'all the poverty and all scientific discoveries; for these are the work of the piteously-brutalised multitudes also. I think I follow your meaning. . . . You would give them a taste also for political and social speculation, for art, for literature, and languages, possibly for the masterpieces of modern Parisian fiction.'" Like all people possessed of hobbies outside of which, is no salvation, Mrs. Norham was innocent of any sense of humour. "She neither understood nor detected badinage."

Notwithstanding his inherent pessimism, Lacy, on the accession of his fortune, which did not in any degree spoil him, and the bulk of which was spent in restoring the fading glories of the family estate, managed to find life very bearable. In a chateau on the Mediterranean he entertains a charming and exclusive party of guests. The little love idyll that is played there finds a fitting frame in this ideal environment.

Here we must leave our hero, trusting that those readers to whom his character and the problems touched upon may prove interesting will secure speedily Mr. Mallock's latest addition to the philosophical school of fiction, of which he is so able and gifted an exponent.

* "The Individualist." By W. H. Mallock. Publishers, Chapman and Hall (Limited), London. 6s.

### The Nurses' Bookshelf.

[We invite Correspondence, Criticism, Enquiries, and Notes on Books likely to interest Women and Nurses. Address, Editor, THE HOSPITAL (Nurses' Book World), 28 & 29, Southampton Street, Strand, London, W.C.]

THE AMERICAN REVOLUTION. Part I., 1766-1776. By the Right Hon. Sir GEORGE OTTO TREVELYAN, Bart., author of "The Life and Letters of Lord Macaulay," &c. (Longmans.)

IN these days, when there is so much cordiality in the relations existing between John Bull and Uncle Sam, and most things American commend themselves to English tolerance, Sir George Trevelyan's history of the conflict between Great Britain and her North American colonies will probably be read by many, including, as well as the student of history, the general reader, who will find pleasure in the author's fresh, graphic style and picturesque elaboration of details, in addition to the interest of the subject matter. Perhaps English readers will not relish the contrast which is drawn between the state of American society just before the war of independence and that of the corresponding classes in the mother country, for the result of the comparison does not redound to our credit. In the description given by Sir George Trevelyan all the features of American life and character are idealised—the blemishes are omitted, and one is left to forget that slavery was recognised by law in most of the much-vaunted commonwealths, and that a great part of the population suffered degradation and too often cruelty as slaves to the other part. The loyalty of the colonists to the mother country we also think is exaggerated, and the utmost is made of the blunders of the home Government. They were dire and incredible enough, as we know, but some allowance should be made for the prejudices of the age. In spite of its evident bias in favour of our American cousins, however, and its occasional over-discursiveness, the book is intensely interesting and readable, and few will lay down the first instalment of this history of the famous struggle without eagerly anticipating another volume.

THE CARE OF THE DYING. A Lecture to Nurses. By OSWALD BROWNE, M.A., M.D., F.R.C.P. (George Allen, Ruskin House. Price 6d.)

THIS little pamphlet is one which with advantage might be placed in the hands of those who minister to the sick. We are accustomed to observe changes for the better or worse in all cases of illness, but less thought is given to what is usually known as the phenomenon of dying. There are so many little things that conduce to the patient's comfort and peace of mind if only the nurse would take the trouble to understand them. The hints given by Dr. Browne, for instance, with regard to the administration of nourishment are admirable. The question as to when the effort to feed should be abandoned has often puzzled many a thoughtful nurse. Thus he says: "We may take it as a rule in our administration both of food and stimulants that they should be continued only so long as the lips close upon them and an act of swallowing follows promptly. When liquids merely trickle down the throat, and after a time only excite a faint effort of swallowing, they should no longer be persisted in" (p. 37). It is comforting also to know that the actual process of dying is by no means a painful one, however distressing it may be to the friends. The functions of the nerve centres in which the consciousness of pain resides are the first to suffer, owing to the impoverishment or impurity of the blood supply, the actual agony or struggle being merely mechanical. Dr. Browne rightly dwells on the importance of quietness and repose, and the attention that should be paid to the spiritual needs of the patient. Well indeed would it be if every nurse would strive to understand the practical hints thrown out by this little work, and endeavour to carry them out when opportunity arises.

THE POCKET CASE BOOK. For District and Private Nurses. (The Scientific Press, Limited, 28 and 29, Southampton Street, W.C. Price 1s.)

THIS case book has been compiled by a physician in the hope that it will be found useful by trained nurses. It is designed to ensure accuracy on the nurse's part, memory at best being an unsatisfactory and treacherous faculty to rely on in our dealings with the sick. On one side of the page is a printed list of every function a nurse is expected to report on, with a blank ruled space opposite for her entries. On the opposite page are spaces for the date, morning and evening temperature, pulse, and respiration. The headings are arranged with great clearness, and, in spite of the small space, the information is so tabulated as to embrace every detail and induce accuracy of observation. We have much pleasure in recommending it as a most useful adjunct to the outfit of both district and private nurses.

THROUGH THE LONG NIGHT WATCH. A Manual for Night Nurses. By M. L. B. SOWAT. ("The Church Shop," Commercial Road, E. Post free, 7d.)

THIS is a collection of prayers, ejaculations, and verses that are intended to help nurses in their spiritual difficulties. Fatigue often depresses and produces a state of mental strain which is most difficult to fight against. A rightly-balanced mind is not easy of attainment when the body is worn out or below par. It is at such times that the watcher's greatest consolation is found in religious exercises, and in this little book are many beautiful and comforting thoughts applicable to all occasions. Especially at the time of death, and afterwards, will the short offices contained therein be appreciated.

### Appointments.

MOGDEN ISOLATION HOSPITAL, ISLEWORTH.—Miss L. Snow has been appointed Matron. She was trained at St. George's, Hanover Square, and has since worked at Brook Hospital, Croydon Hospital, and Mogden Isolation Hospital.

IPSWICH ISOLATION HOSPITAL.—Miss M. Mumford has been appointed Matron. She was trained at the General Hospital, Birmingham, and has since been charge nurse at the South-Eastern Hospital, New Cross.

MONSAL FEVER HOSPITAL, MANCHESTER.—Miss A. Cox, who has been lady superintendent at the Hull Royal Infirmary during the last eight years, has been appointed Superintendent of Nurses at the above hospital.

### Presentations.

ON Saturday, August 5th, Nurse Martin, one of the Queen's Jubilee Nurses, was presented with a very handsome testimonial by many of her patients and friends in the Darwen district, where she had been superintendent nurse upwards of seven years. The present consisted of a silver tea service and illuminated address, the latter signed by the medical men of the town. The presentation was made privately, but the number of names appended to the address showed how generally popular Nurse Martin was in the district, and how thoroughly she had earned the good-will of those with whom she came in contact.

### HOLIDAYS FOR OVERWORKED NURSES.

A LADY generously places a room and board free to any nurse who needs a holiday after September 1st for a fortnight. Two friends might go together, as the room has a double bed. The nurses will be treated in every way as friends, and will be considered in that light. Any nurse wishing to avail herself of this kind offer must communicate with the "Travel Editor," and forward two references as to personal character.

### Everybody's Opinion.

[Correspondence on all subjects is invited, but we cannot in any way be responsible for the opinions expressed by our correspondents. No communication can be entertained if the name and address of the correspondent is not given, as a guarantee of good faith but not necessarily for publication, or unless one side of the paper only is written on.]

### DO NURSES SMOKE?

"A PATIENT" writes: I feel very strongly on the subject of private nurses smoking. "M. G. S." says that the effacing of the smell of smoke is a small affair, but I doubt if a delicate person would consider it effaced when a stronger one was satisfied on that point. The inclination to nausea in so many internal cases causes the patient to be intensely sensitive to any strong or irritating scent, and the odour of tobacco, however faint, would be extremely unpleasant. Also, if a nurse were indulging in a cigarette, "at the right time and in the proper place," what would be the result if for some special and unforeseen reason the patient suddenly called for her assistance? Besides this, it is well known that the habit of smoking is one that is very difficult to keep within bounds, and if a nurse limits herself for awhile to one cigarette a day—and one only—it is more than likely she may find other occasions on which she feels herself justified in repeating the enjoyment, and so gradually her strict rule will be relaxed, and she will develop into a constant smoker, and her patient will suffer accordingly.

"MARIAN" writes: As one who has held the post of sister in three hospitals, perhaps I may be allowed to reply to M. G.'s question. It has been wisely said by someone: "We live in advanced times; how few deserve the beautiful name of woman." We are surrounded on every side by women. Women of fashion and society, women of business, the literary woman, the nurse, &c. Of all these the nurse is by far the most conspicuous, and, in many ways, has the highest duties to perform, for she has the care of the sick, the convalescent, and, above all, the dying. Surely, then, it behoves the nurse to be true to her calling, and in one word, to be a *woman*. All ought to look up to her. We want to rise to her level, not to be dragged down to the level of the woman who tries to walk in the footsteps of man. I am well aware that some doctors agree that "when nerves are overstrung the effect of a cigarette is of immediate and soothing relief," but it is also true that when nerves are overstrung a good tonic, a few hours off duty, or the reading of an interesting book will also bring relief. Man ought to look up to woman as the gentler nature, and not to feel that the woman whose work is dedicated to God's service is trying to "act the man." Now for the last point, which shall also be a question. Does not smoke only too often lead to drink? I am a nurse of many years' standing, and am not old enough to be considered old-maidish, but I never knew anyone who in their heart really respected women smokers. Surely a good run in the open-air, or, if possible, a drive or ride on a 'bus, does far more to refresh us and to brighten our spirits for our patients than the "loose" talk which invariably accompanies the woman smoker.

### NURSES' DRESS.

"M. C." writes: While quite agreeing with "Vera's" remarks upon the abuse of uniform by the women one sees daily abroad in muslin aprons, high-heeled buckled shoes, veils nearly two yards long, fringes, jewellery, and silver chatelaines jangling with articles that no nurse would ever require under any circumstances, I think that any intelligent person who gave a thought to the matter could easily distinguish the genuine nurse from the made-up article. But an equally grave abuse, and one not so easy of detection, is the wearing of orthodox uniform by unqualified women, who in nine cases out of ten are ignorant of the primary principles of a nurse's work; *e.g.*, young "pros." scarcely a month in hospital will spend all their first few shillings of salary in an up-to-date cloak and bonnet with a long veil. Domestic nurses will do likewise, labouring under an idea that hospital uniform gives them a more refined appearance, "assistant nurses" at infectious hospitals with, perhaps, six weeks' experience, and many more types of the untrained "nurse," all rush into uniform on the earliest opportunity, to the great annoyance of thoroughly trained and certificated experienced women, who, in my opinion, are the only ones entitled to wear it. Should a street accident occur and one of these wearers of bonnet and cloak be pushed to the front of the inevitable crowd, what would she do? More likely than not, either by an exhibition of hopeless ignorance or by well-meant but ill-judged treatment of the sufferer, bring discredit and contempt upon the profession of which she was supposed, by virtue of her costume, to be an efficient member. The uniform of soldiers, sailors, and policemen is legally protected, and every nurse will, I am sure, rejoice to see the day when similar protection is accorded to the bonnet and cloak which are now so abused by those who have no genuine right to wear them.

### BREAKFAST IN BED.

"ONE WHO LEARNS TO LIVE" observes: When nurses are nursing in private houses they must remember that they are there not to enjoy themselves, but to work. Short-tempered nurses—and there are not a few doing private work—are liable to do rash things, and the saying, "Look before you leap," never applies better to anyone than to the private nurse. Ladies, as a rule, have good opinions of nurses, and they estimate their positions as being higher than that of general servants, and for this, as well as many other reasons, the nurse does not have her breakfast in the kitchen. Of course no private nurse would think of having her breakfast with the family in the breakfast-room. Very often separate rooms are not available when a member of a family is ill, and the nurse is obliged to take her meals in the bedroom. To grumble under such circumstances would be very bad breeding indeed. On the other hand, when there are plenty of spare rooms, no lady would even think of giving a nurse her meals in the bedroom. But to attempt to teach a woman who is not a lady is almost a matter of impossibility; besides, it does not come within the scope of a nurse's duties. If ever again "One Who Lives to Learn" meets with unreasonable treatment she had better speak to her patient's doctor, and a word from him will be like oil on troubled waters. I have been many a time glad of a breakfast regardless of the the place wherein I ate it.

"ONE WHO LIVES TO LEARN" replies to some of her critics as follows: I am much obliged for the two answers to my question. It is true I should have preferred more than two, but one cannot have everything. I quite expected a reply on the lines of—especially—that of "An Old Nurse," because I have noticed that if anyone raises a protest, however tentatively, they seldom escape being set to rights by someone with a superior "love" and, clearly, fitness, "for the work." Sometimes a text is added to the rebuke and homily, but I have been spared the text. "Edith," whose experience has been rather unfortunate, and who has evidently grown frankly tired of—if I may use the expression—"pigging it," puts her finger on the spot when she says she was not thought to require any other room. A few years ago governesses were not thought to require common civility or decent treatment. Now it is the nurse who is thought to be quite "reasonably" considered if she is asked to sleep, wash, dress, and take her food in the patient's room. "An Old Nurse" must have seen by my context that I was not speaking of the very rare occasions when one's work obliges one, as anyone's work might do at times, to live without the most ordinary accommodation. I have no objection whatever to taking my meals with the maid-of-all-work, or the young man from the shop, if it is more convenient to anyone concerned. I may add that I have done both; while liveried servants have no charms for me. But to be forced to eat in a small unaired room in which two adults—one as likely as not sick to some extent—have been breathing for nine or ten hours is a different thing altogether. One of the two is homely. The other is, I think, more or less revolting, and often unnecessary more than less.

### Wants and Workers.

WILL some charitable nurse be kind enough to send her copy of THE HOSPITAL (after she has read it) to a poor nurse at Parochial Hospital, Carrickmacross, Co. Monaghan?

### For Reading to the Sick.

"NOT AS I WILL, BUT AS THOU WILT."

—*Mark* xiv. 36.

THY way, not mine, O Lord,
However dark it be;
Lead me by Thine own hand,
Choose out the path for me.
Smooth let it be or rough,
It will be still the best;
Winding or straight, it leads
Right onward to Thy rest.

Take Thou my cup, and it
With joy or sorrow fill
As best to Thee may seem.
Choose Thou my good and ill;
Choose Thou for me my friends,
My sickness or my health;
Choose Thou my cares for me,
My poverty or wealth.

Not mine, not mine the choice
In things or great or small;
Be Thou my guide, my strength,
My wisdom and my all. —*Bonar.*

**Reading.**

"Thy will be done." Now mark the emphasis on *done.* He prays that God's will be done. It is not that God's will be borne, endured, put up with. There is activity in his prayer. It is not mere resignation. How often is this prayer toned off into mere endurance, sufferance, passivity. "Thy will be done," people say resignedly. "There is no help for it. We may just as well submit. God evidently means to have His way. Better to give in at once and make the best of it." Well, this is far from the ideal prayer. It may be nobler to suffer God's will than to do it; perhaps it is. But there is nothing noble in resignation of this sort—this resignation under protest as it were. And it disguises the meaning of the prayer, "Thy will be done." It is intensely active. It is not an acquiescence simply in God's dealing. It is a cry for more of God's dealing—God's dealing with me, with everything, with everybody, with the whole world.

The ideal man's only chance of happiness, of usefulness, of work, is to join the living rill of his will to that. . . . He will be among the forces and energies and powers, that he may link his weakness with God's greatness, and his simplicity with God's majesty, that he may become a force, an energy, a power for duty and God. Perhaps God may do something with him. Certainly God will do something in him, for it is God Who worketh in him both to will and to do of His good pleasure. So his one concern is to be kept in the will of God.

The ideal man does not want a bed of roses, or his pathway strewn with flowers. He wants to do God's will. . . . Sickness or poverty, just what God sends. . . . He does not want his friends to live, himself to live or die. God's will be done.

The currents of his life flow far below the circumstances of things. There is a deeper principle in it than to live to gratify himself. And so he simply asks that in the ordinary round of his daily life there may be no desire of his heart more deep, more vivid, more absorbingly present than this, "Thy will be done."

Lord Jesus, as Thou wilt! If among thorns I go,
Still sometimes here and there let a few roses blow.
No! Thou on earth the thorny path hast gone,
Then lead me after Thee, my Lord; Thy will be done.
—*Schmolk.*

—*From Drummond's "Ideal Life."*

### Notes and Queries.

**The contents of the Editor's Letter-box have now reached such unwieldy proportions that it has become necessary to establish a hard and fast rule regarding Answers to Correspondents. In future, all questions requiring replies will continue to be answered in this column without any fee. If an answer is required by letter, a fee of half-a-crown must be enclosed with the note containing the enquiry. We are always pleased to help our numerous correspondents to the fullest extent, and we can trust them to sympathise in the overwhelming amount of writing which makes the new rules a necessity.**

**Every communication must be accompanied by the writer's name and address, otherwise it will receive no attention.**

*Queen's Nurses.*

(181) Will you tell me where Queen's nurses are sent for midwifery training in England besides Cheltenham?—*R. P.*

Apply to the Secretary, Q.V.J.I., and Queen Katherine's Hospital, Regent's Park, N.W.

*Terms for Training.*

(182) Will you kindly inform me what terms are required for a probationer at the Diamond Jubilee Hospital at Woodford? That is to say, is a premium required; and, if not, what salary is granted for the first year's service? Also, how long a time is it necessary to serve, and is a certificate granted?—*L. B.*

Why not write direct to the matron? She will, of course, give you all the information you require on the subject. May we draw your attention to the importance of selecting a training school approved by the Local Government Board if you contemplate becoming a nurse? Otherwise you will find that, however well taught you may have been, your training will not be recognised by the chief nursing authorities.

*Holiday Home.*

(183) I beg to ask you if you have a letter for a home of rest you could give me? I have three weeks' leave of absence, but I cannot afford to pay for a holiday, and I have been told that you have letters to dispose of.—*Night Nurse.*

There appears to be some little confusion here. The Hospital Convalescent Fund helps nurses recovering from illness to rest and change, but not nurses needing a holiday only. Miss Cole, 188, Ebury Street, S.W., has compiled a list of holiday homes for the Y.W.C.A. girls, which is sent post free on application for 1½d.

*Hearthstone.*

(184) A correspondent very kindly writes recommending the use of the lump hearthstone in preference to the white squares in order to produce a good effect on stone steps and corridors. She is also emphatic as to the necessity of thoroughly washing off the soiled stone before rubbing on a plentiful quantity of fresh stone.

*Consumption.*

(185) Can you tell of an open-air sanatorium for consumption suitable for a working man of 21? He could afford a small payment.—*Inquirer.*

The Royal National Hospital for Consumption at Ventnor adopts the open-air treatment. Admission, by subscriber's letter free; or by payment of 10s. a week.

*R.B.N.A.*

(186) I have just finished three years' hospital training, and I want to know where I should register as a trained nurse. Also, I want to belong to the Royal British Nurses' Association. Could you give me the address?—*Augusta.*

The Secretary, R.B.N.A., 17, Old Cavendish Street, W., will give you all particulars. This is the only society that registers trained nurses.

*St. John Ambulance.*

(187) Will you kindly tell me if there are any ambulance classes for girls in Birmingham?—*Miss S.*

Write to the Secretary, St. John Ambulance Association, St. John's Gate, Clerkenwell, E.C., and ask what classes have been arranged for Birmingham.

*Mental Cases.*

(188) I wish to start a "home" for slight mental cases. Will you tell me how many I may take in without procuring a licence? Please advise what steps to take if a licence is necessary.—*K. K.*

It is a very risky thing to start a home for "slight mental cases." It is entirely illegal to take charge of, receive to board or lodge, or to detain a lunatic or alleged lunatic for payment, except when fully certified in accordance with the provisions of the Lunacy Act; and except by the special permission of the Commissioners it is illegal for anyone to receive more than one patient, even when so certified, except in a licensed institution. On the other hand, the lunacy law has nothing whatever to do with such people as are not lunatics, and you may think that it is a simple matter to arrange to receive only such "slight mental cases" as are not lunatics or persons of unsound mind. It is not, unfortunately, so simple as you may think, for if any of these slight mental cases should end by becoming really insane, you would have great difficulty in showing that you had not been taking charge of a lunatic for payment, which is a misdemeanour, and renders you liable to a fine of £50. It is the uncertainty of the boundary line, and the fact that among slight mental cases there are always some who are really in the early stages of insanity, and who will in the end become insane, and thus land you in difficulties, that renders the keeping of such a home as you propose a risky business. Plenty of people do it, but the risk is there all the same. As to the steps necessary to set up a licensed house, you would have to apply to the Lunacy Commissioners, but there is no great readiness shown to increase the number of private asylums, and, moreover, "slight mental cases," *i.e.*, cases not sufficiently severe to be certified, would not be admissible to an asylum. Of course, you will say it is a very serious blot in the law that it is practically impossible, without serious risk, to accommodate such cases—and so it is.

### Travel Notes.

By the Travel Editor.

### XXXIII.—NORTH WALES.

I think that it may be useful for nurses working in the North of England to have some slight account of reasonable places in North Wales in which to spend a short holiday. England will always be dearer than the Continent, but it is not so easy to cross the Channel when living northwards, and the expense of the preliminary journey south counterbalances the extra charge for board and lodging.

### Conway or Llanrwst.

Conway is not an expensive place, and is a most interesting old city, especially to those with artistic tastes. It is a very good centre for excursions, and as such I think somewhat overlooked. It is about twenty miles from Bettws-y-Coed, an excursion easily managed in a day. Llandudno is close by, and Penmaenmawr very attainable. Both Llandudno and Penmaenmawr are expensive. Lodgings in Conway would be much cheaper, and these beautiful but tourist-ridden spots can be visited constantly from the old city. The bathing at Conway is not good, and should be carried out in those more favoured spots. The castle and walls of the town are full of interest; altogether the effect is somewhat that of a foreign town. You must also visit the church; the venerable tower is a great feature of the town. There is a stone, too, in memory of a worthy person who was the forty-first child of Nicholas Hookes, and, not content with that distinction, he is further stated to be the father of twenty-seven children. A rather more romantic interest attaches to the fact that the churchyard is associated with Wordsworth's "We are seven." Near by is what is called "The Great House," built in the sixteenth century: it is now used as the Cambrian Academy. For those who do not care for town life, and yet cannot stand the charges at Bettws-y-Coed and such well-known places, Llanrwst offers attractions; it lies two-thirds on the road to Bettws-y-Coed, and in the loveliest scenery of the Conway Valley. From here you can visit Capel Curig and the Snowdon district, Bettws-y-Coed, and Festiniog. For myself, I think it an infinitely more agreeable place in which to stay than those very popular places, where not only are charges high, but there is not a moment's peace from touts, itinerant vendors of all sorts of useless articles, and everlasting calls for small fees before one can enjoy the beauties of nature.

### Colwyn Bay.

Living here, too, is not dear; there is a temperance hotel and some few boarding-houses. The climate is very mild and adapted for a late holiday. The best bathing is at Llandrillo-yn-Rhos, which is a kind of suburb of Colwyn. The old church of Llandrillo is interesting to archæologists, and extremely ancient. The many surrounding walks are the principal attraction of Colwyn; they are almost endless, but you must find them out for yourselves, for my space is limited. Longer excursions may be made to Abergele, Rhyl, Rhuddlan, and St. Asaph. The castle at Rhuddlan is worth a visit, admission only twopence. As well as being an unusually strong fortress Rhuddlan contained within its walls a Dominican priory. There is nothing to see at St. Asaph except the cathedral, which is shut except at service time—eight a.m. and three p.m. At other hours the verger must be sought; his address is on the gate.

### Corwen makes another very good and inexpensive centre; it is considerably to the south of Ruthin, and from it you will be able to explore the beautiful little Vale of Clwyd. Bettws-y-Coed is also reached easily by coach, and through most lovely scenery. To Bala is a good coach and cycle road, but Bala itself is not quite such a good centre.

### Llanberis and Snowdon.

No doubt Llanberis is one of the most pleasing spots in North Wales, but from its convenience for mountaineering generally, and its vicinity to Snowdon, it is not cheap; still, if visited out of the season, terms may be arranged reasonably, but prices will far exceed those at the other places I have mentioned. The Pass of Llanberis is rightly considered the finest in Wales. Between the north side of Snowdon and the side of Glyder-fawr its wild grandeur is enchanting. It is about six miles long, and since the making of the road carriages can go all the way. The easiest ascent of Snowdon is from the Llanberis side, and from here, too, starts the Mountain Railway. This was opened in 1896, and signalised the event by a serious accident, resulting in the death of one passenger and the wrecking of two trains. All has now been remedied, and it works with the precision of the Swiss mountain lines.

### Hints to Travellers.

It is very important not to multiply impedimenta on a journey, but all the same my advice is, never travel without a tea basket. Now these useful articles, as supplied by the trade, are very expensive luxuries, but I fitted up my own, and it has travelled many thousands of miles with me. Ask your grocer or wine merchant for one of the ordinary square baskets in which they send out half dozens of champagne. It is about fourteen inches by ten, closes with two wicker loops and a stick, and has a firm handle at the side. Then buy two plates, two cups, and two saucers of enamelled ware, with two common forks, spoons, and knives; then take strong white cotton elastic and make slips to hold the plates and saucers round the sides of the basket and small ones on the inside of the lid for the knives, forks, and spoons. Buy a spirit kettle and lamp that folds into the smallest compass. Let the kettle have a screw lid and cover for the spout so that it may be carried full of water. Have a small tin to carry as much tea as you are likely to require on any one journey, a bottle for milk, a small tin box for wax matches, and a supply of grease-proof paper to wrap provisions in. With this equipment wherever you are stranded you will not be in despair nor dependent on the fitful chances of railway buffets.

### TRAVEL NOTES AND QUERIES.

Brunnen (Klopstock).—It is rather full in the season, and it would be well to write for rooms in advance. Hotel Waldstatter, on the lake, 8 to 12 francs; Hotel Pension Hirsch, on the quay, much humbler, 6 to 7 francs. It is a delightful situation for walks on the lake along the Axenstrasse and for exploring the St. Gothard pass.

Totnes (Architect).—It is still primitive, and there are some delightfully artistic corners left, though its glory is continually departing. It is a very nice spot to stay in if you do not mind the rather enervating climate. Boating up and down the Dart is a constant pleasure. The three hotels—Seymour, Seven Stars, and the Castle—are not expensive; but if you remain as long as a week lodgings would be better. Berry Pomeroy, Staverton, and Dartington are all close, and you are speedily on Dartmoor.

Bay of St. Malo (Matteo).—If economy is an object go to Miss Humphreys, Rue de Pomellec, St. Servan, or to Miss Dixon, Place Constantine; or, in Paracué, to the Hotel Continental, from 6 francs; or the Hotel des Bains, from 6 francs. Dinard is nearly twice as expensive, and you can enjoy all its advantages without living there. Steamers cross to and fro from St. Malo and St. Servan every half-hour. Mont St. Michel can be visited in a day, though it is better to go from Saturday to Monday and so see Dol on market day.

Board in a French Family (Domestic).—This is almost unknown. The French are averse to taking in strangers. A few French pastors take pupils, but then your object is defeated, because there are other English boys. It is not a very safe life for a boy of your son's age. Your best plan would be to advertise in a French newspaper, but I think it is a risky thing altogether without some knowledge of the parties.

Valescuse (Portsmouth).—I am away from home and have no lists of hotels and pensions with me. You do not wish to leave England just now, so I should advise you to write to me again after September 8th, when I shall be again in England. St. Raphael is not very expensive, nor is Bordighera. Valescuse I do not know so well, but will find out. You should not settle upon any place without the advice of your doctor, the climate of the Riviera varies so much. Ask him how Pau in the Basses Pyrénées would do. I think I might arrange something reasonable there for you. If you can wait remember September 8th, if not I will do my best before, but I am far from posts in the Highlands.

